# Approximation for the gamma function via the tri-gamma function

**DOI:** 10.1186/s13660-017-1585-7

**Published:** 2017-12-16

**Authors:** Xu You, Xiaocui Li

**Affiliations:** 1 Department of Mathematics and Physics, Beijing Institute of Petrochemical Technology, Beijing, 102617 P.R. China; 2 School of Science, Beijing University of Chemical Technology, Beijing, 100029 P.R. China

**Keywords:** 33B15, 26D15, 41A25, approximation, gamma function, inequalities, multiple-correction method

## Abstract

In this paper, we present a new sharp approximation for the gamma function via the tri-gamma function. This approximation is fast in comparison with the recently discovered asymptotic series. We also establish the inequalities related to this approximation. Finally, some numerical computations are provided for demonstrating the superiority of our approximation.

## Introduction

It is well known that we often need to deal with the problem of approximating the factorial function *n*! and its extension to real numbers called the gamma function, defined by

$$ \Gamma (x)= \int_{0} ^{\infty }{t^{x-1}e^{-t}}\,dt,\quad \operatorname{Re}(x)>0, $$

and the logarithmic derivatives of $\Gamma (x)$ are called the psi-gamma functions, denoted by

$$ \psi (x)=\frac{d}{dx}\ln \Gamma (x)=\frac{\Gamma '(x)}{\Gamma (x)}. $$

For $x > 0$, the derivatives $\psi '(x)$ are called the tri-gamma functions, while the derivatives $\psi^{(k)}(x)$, $k = 1, 2, 3,\ldots$ , are called the poly-gamma functions.

Mortici [1] proved that

1.1 $$\begin{aligned} \Gamma (x+1)=\sqrt{2\pi x} \biggl( \frac{x}{e} \biggr) ^{x} \exp \biggl( \frac{1}{12} \psi '(x+1/2) \biggr) \exp h(x) \end{aligned}$$

and

1.2 $$\begin{aligned} h(x)=\sum_{m=1}^{\infty } \frac{B_{2m}}{2m(2m-1)x^{2m-1}} -\sum _{m=1} ^{\infty } \frac{B_{m-1}}{12(x+1/2)^{m}}, \end{aligned}$$

where $B_{k}$, $k\geq 0$, noting the Bernoulli numbers which are generated by

1.3 $$\begin{aligned} \frac{z}{e^{z}-1}=\sum_{k=0}^{\infty }B_{k} \frac{z^{k}}{k!}. \end{aligned}$$

It is found that

1.4 $$\begin{aligned} h(x)=\frac{1}{240x^{3}}-\frac{11}{6720x^{5}}+\frac{107}{80{,}640x^{7}}- \frac{2911}{1{,}520{,}640x ^{9}}+O \biggl( \frac{1}{x^{11}} \biggr) . \end{aligned}$$

However, those coefficients of the asymptotic formula (1.1) are not complete. The asymptotic expansion of $\Gamma (x+1)$ via the tri-gamma function can be generalized to the general cases by the arguments in [2] as follows.

Barnes (1899) and Rowe (1931) have shown that

1.5 $$\begin{aligned} \begin{aligned}[b] \ln \Gamma (z+a)&= \biggl(z+a-\frac{1}{2}\biggr)\ln z-z+ \frac{1}{2} \ln 2\pi \\ &\quad{} +\sum_{k=1}^{n} \frac{(-1)^{k+1}B_{k+1}(a)}{k(k+1)}z^{-k}+O\bigl(z^{-n-1}\bigr), \end{aligned} \end{aligned}$$

where $\vert \arg z\vert \leq \pi -\varepsilon $, $\varepsilon >0$ and $B_{k}(x)$ is the Bernoulli polynomial. If $a=\frac{1}{2}$, $B_{k}(a)$ vanishes if *k* is odd, note that

1.6 $$\begin{aligned} \ln \Gamma \biggl(z+\frac{1}{2}\biggr)=z(\ln z-1)+\frac{1}{2} \ln 2\pi +\sum_{k=1} ^{n}\frac{B_{2k}(\frac{1}{2})}{2k(2k-1)}z^{1-2k}+O \bigl(z^{-2n-1}\bigr) \end{aligned}$$

for $\vert \arg z\vert \leq \pi -\varepsilon $, $\varepsilon >0$ listed in [2], p. 32, (5). We can get as $x\rightarrow \infty $,

1.7 $$\begin{aligned} \ln \Gamma \biggl(x+\frac{1}{2}\biggr)=x\ln x-x+\frac{1}{2} \ln 2 \pi +\sum_{k=1} ^{\infty }\frac{B_{2k}(\frac{1}{2})}{2k(2k-1)} \frac{1}{x^{2k-1}}. \end{aligned}$$

So we consider a function $h(x)$ defined by

1.8 $$\begin{aligned} \Gamma (x+1)=\sqrt{2\pi } x^{x+1/2} e^{-x} \exp \biggl( \frac{1}{12} \psi ' \biggl( x+\frac{1}{2} \biggr) \biggr) \exp h(x). \end{aligned}$$

By (1.7), one can easily obtain that as $x\rightarrow \infty $,

$$\begin{aligned} h(x) &=\ln \Gamma (x+1)-\frac{1}{2} \ln 2\pi - \biggl( x+ \frac{1}{2} \biggr) \ln x +x -\frac{1}{12}\psi ' \biggl( x+\frac{1}{2} \biggr) \\ &=\sum_{n=1}^{\infty }\frac{B_{2n}}{2n(2n-1)} \frac{1}{x^{2n-1}}- \frac{1}{12}\frac{d^{2}}{dx^{2}}\ln \Gamma \biggl( x+ \frac{1}{2} \biggr) \\ &=\sum_{n=1}^{\infty } \biggl( \frac{B_{2n+2}}{2(n+1)(2n+1)}+\frac{(1-2^{1-2n})B _{2n}}{12} \biggr) \frac{1}{x^{2n+1}}. \end{aligned}$$

Thus, together with (1.8) the asymptotic expansion can be explicitly expressed as

1.9 $$\begin{aligned} \Gamma (x+1)=\sqrt{2\pi } x^{x+1/2} \exp \Biggl( \frac{1}{12}\psi ' \biggl( x+\frac{1}{2} \biggr) -x+\sum _{n=1}^{\infty } \frac{c_{n}}{x^{2n+1}} \Biggr) , \quad x\rightarrow \infty , \end{aligned}$$

where

1.10 $$\begin{aligned} c_{n}=\frac{B_{2n+2}}{2(n+1)(2n+1)}+\frac{(1-2^{1-2n})B_{2n}}{12}, \end{aligned}$$

here $B_{n}$ denotes the Bernoulli number.

In this paper we will apply the *multiple-correction method* [3–5] to construct a new asymptotic expansion for the factorial n! and the gamma function via the tri-gamma function.

### Theorem 1

*For every integer* $n\geq 1$, *we have*

1.11 $$\begin{aligned} \Gamma (n+1)\sim \sqrt{2\pi n} \biggl( \frac{n}{e} \biggr) ^{n} \exp \biggl( \frac{1}{12}\psi '(n+1/2) \biggr) \exp \bigl( \eta_{1}(n)+\eta_{2}(n) \bigr) , \end{aligned}$$

*where*

$$ \eta_{1}(n)=\frac{\frac{1}{240}}{n^{3}+\frac{11}{28}n}, \quad\quad \eta_{2}(n)= \frac{ \frac{193}{282{,}240}}{n^{7} + \frac{108{,}338}{44{,}583} n^{5} - \frac{21{,}252{,}897{,}179}{59{,}061{,}418{,}416} n^{3} + \frac{997{,}042{,}514{,}542{,}183}{188{,}081{,}086{,}945{,}752} n}. $$

Using Theorem 1, we provide some inequalities for the gamma function.

### Theorem 2

*For every integer* $n>1$, *the following holds*:

1.12 $$\begin{aligned} \exp \eta_{1}(n)< \frac{n!}{\sqrt{2\pi n} ( \frac{n}{e} ) ^{n} \exp ( \frac{1}{12}\psi '(n+1/2) ) } < \exp \bigl( \eta _{1}(n)+\eta_{2}(n) \bigr) . \end{aligned}$$

To obtain Theorem 2, we need the following lemma which was used in [6–8] and is very useful for constructing asymptotic expansions.

### Lemma 1

*If the sequence* $(x_{n})_{n\in \mathbb{N}}$ *is convergent to zero and there exists the limit*

1.13 $$\begin{aligned} \lim_{n\rightarrow +\infty }n^{s}(x_{n}-x_{n+1})=l \in [-\infty ,+ \infty ] \end{aligned}$$

*with* $s>1$, *then*

1.14 $$\begin{aligned} \lim_{n\rightarrow +\infty }n^{s-1}x_{n}=\frac{l}{s-1}. \end{aligned}$$

Lemma 1 was proved by Mortici in [6]. From Lemma 1, we can see that the speed of convergence of the sequences $(x_{n})_{n\in \mathbb{N}}$ increases together with the values *s* satisfying (1.13).

## Proof of Theorem 1

*(Step 0) The initial-correction.* We can introduce a sequence $(u_{0}(n))_{n\geq 1}$ by the relation

2.1 $$\begin{aligned} n!=\sqrt{2\pi n} \biggl( \frac{n}{e} \biggr) ^{n} \exp \biggl( \frac{1}{12} \psi '(n+1/2) \biggr) \exp u_{0}(n), \end{aligned}$$

and to say that an approximation $n! \sim \sqrt{2\pi n} ( \frac{n}{e} ) ^{n} \exp ( \frac{1}{12}\psi '(n+1/2) ) $ is better if the speed of convergence of $u_{0}(n)$ is higher.

From (2.1), we have

2.2 $$\begin{aligned} u_{0}(n)=\ln n!-\frac{1}{2}\ln {2\pi n}-n \ln \frac{n}{e}- \frac{1}{12}\psi '(n+1/2). \end{aligned}$$

For any integer *k*, $x > 0$, we have $\psi^{(k)}(x+1)=\psi^{(k)}(x)+(-1)^{k}\frac{k!}{x ^{k+1}}$ and when $k=1$, $x=n$, it yields $\psi '(n+1)=\psi '(n)-\frac{1}{n ^{2}}$. Thus,

2.3 $$\begin{aligned} \begin{aligned}[b] u_{0}(n)-u_{0}(n+1)&= \ln \frac{1}{n+1}- \frac{1}{2}\ln {2\pi n}-n \ln \frac{n}{e} \\ &\quad{} +\frac{1}{2}\ln {2\pi (n+1)} +(n+1) \ln \frac{n+1}{e} - \frac{1}{12} \frac{1}{(n+1/2)^{2}}. \end{aligned} \end{aligned}$$

Developing (2.3) into power series expansion in $1/n$, we have

2.4 $$\begin{aligned} u_{0}(n)-u_{0}(n+1)=\frac{1}{80}\frac{1}{n^{4}}+O \biggl( \frac{1}{n^{5}} \biggr) . \end{aligned}$$

By Lemma 1, we know that the rate of convergence of the sequence $(u_{0}(n))_{n\geq 1}$ is $n^{-3}$.

*(Step 1) The first-correction.* We define the sequence $(u_{1}(n))_{n\geq 1}$ by the relation

2.5 $$\begin{aligned} n!\sim \sqrt{2\pi n} \biggl( \frac{n}{e} \biggr) ^{n} \exp \biggl( \frac{1}{12} \psi '(n+1/2) \biggr) \exp \eta_{1}(n) \exp u_{1}(n), \end{aligned}$$

where

$$ \eta_{1}(n)=\frac{a_{1}}{n^{3}+b_{2} n^{2}+b_{1} n+b_{0}}. $$

From (2.5), we have

2.6 $$\begin{aligned} \begin{aligned}[b] u_{1}(n)-u_{1}(n+1)&= \ln \frac{1}{n+1}- \frac{1}{2}\ln {2\pi n}-n \ln \frac{n}{e} +\frac{1}{2}\ln {2 \pi (n+1)} \\ &\quad{} +(n+1) \ln \frac{n+1}{e}-\frac{1}{12}\frac{1}{(n+1/2)^{2}}- \eta_{1}(n)+ \eta_{1}(n+1). \end{aligned} \end{aligned}$$

Developing (2.6) into power series expansion in $1/n$, we have

2.7 $$\begin{aligned} u_{1}(n)-u_{1}(n+1)&= \biggl( \frac{1}{80}-3a_{1} \biggr) \frac{1}{n^{4}} + \biggl( -\frac{1}{40}+a_{1}(6+4b_{2}) \biggr) \frac{1}{n^{5}} \\ &\quad{} + \biggl( \frac{15}{448}+5a_{1}\bigl(-2 + b_{1} - 2 b_{2} - b_{2}^{2}\bigr) \biggr) \frac{1}{n^{6}} \\ &\quad{} + \biggl( -\frac{17}{448}+a_{1} \bigl(15 + 6 b_{0} + 20 b_{2} + 15 b_{2} ^{2} + 6 b_{2}^{3} - 3 b_{1} (5 + 4 b_{2}) \bigr) \biggr) \frac{1}{n^{7}} \\ &\quad{} +O \biggl( \frac{1}{n^{8}} \biggr) . \end{aligned}$$

By Lemma 1, the fastest possible sequence $(u_{1}(n))_{n\geq 1}$ is obtained as the first four items on the right-hand side of (2.7) vanish.

(i) If $a_{1}\neq \frac{1}{240}$, then the rate of convergence of the sequence $(u_{1}(n))_{n\geq 1}$ is $n^{-3}$.
(ii) If $a_{1}=\frac{1}{240}$, $b_{2}=0$, $b_{1}=\frac{11}{28}$, $b_{0}=0$, from (2.7) we have
  $$\begin{aligned} u_{1}(n)-u_{1}(n+1)=\frac{193}{40{,}320}\frac{1}{n^{8}}+O \biggl( \frac{1}{n ^{9}} \biggr) , \end{aligned}$$
  and the rate of convergence of the sequence $(u_{1}(n))_{n\geq 1}$ is at least $n^{-7}$.

*(Step 2) The second-correction.* So we define the sequence $(u_{2}(n))_{n\geq 1}$ by the relation

2.8 $$\begin{aligned} n!\sim \sqrt{2\pi n} \biggl( \frac{n}{e} \biggr) ^{n} \exp \biggl( \frac{1}{12} \psi '(n+1/2) \biggr) \exp \bigl( \eta_{1}(n)+\eta_{2}(n) \bigr) \exp u_{2}(n), \end{aligned}$$

where

$$ \eta_{2}(n)=\frac{a_{2}}{n^{7}+b_{6} n^{6}+b_{5} n^{5}+b_{4} n^{4}+b _{3} n^{3}+b_{2} n^{2}+b_{1} n+b_{0}}. $$

Using the same method as above, we obtain that the sequence $(u_{2}(n))_{n\geq 1}$ converges fastest only if $a_{2}= \frac{193}{282{,}240}$, $b_{6}=0$, $b_{5}=\frac{108{,}338}{44{,}583}$, $b_{4}=0$, $b _{3}=-\frac{21{,}252{,}897{,}179}{59{,}061{,}418{,}416}$, $b_{2}=0$, $b_{1}= \frac{997{,}042{,}514{,}542{,}183}{188{,}081{,}086{,}945{,}752}$, $b_{0}=0$, and the rate of convergence of the sequence $(u_{2}(n))_{n\geq 1}$ is at least $n^{-15}$. We can get

$$\begin{aligned} u_{2}(n)-u_{2}(n+1)= \frac{168{,}288{,}414{,}443{,}284{,}544{,}502{,}901}{516{,}188{,}874{,}145{,}329{,}388{,}523{,}520} \frac{1}{n ^{16}}+O \biggl( \frac{1}{n^{17}} \biggr) . \end{aligned}$$

The new asymptotic (1.11) is obtained.

## Proof of Theorem 2

The double-side inequality (1.12) may be written as follows:

$$ f(n)=\ln \Gamma (n+1)-\frac{1}{2} \ln 2\pi n -n \ln \frac{n}{e} - \frac{1}{12}\psi '(n+1/2)-\eta_{1}(n)- \eta_{2}(n)< 0 $$

and

$$ g(n)=\ln \Gamma (n+1)-\frac{1}{2} \ln 2\pi n -n \ln \frac{n}{e} - \frac{1}{12}\psi '(n+1/2)-\eta_{1}(n)>0. $$

Suppose $F(n)=f(n+1)-f(n)$ and $G(n)=g(n+1)-g(n)$. For every $x>1$, we can get

3.1 $$\begin{aligned} F''(x)=\frac{A_{2}(x-1)}{70 x^{3} (1 + x)^{3} (1 + 2 x)^{4} (11 + 28 x ^{2})^{3} (39 + 56 x + 28 x^{2})^{3} \Psi_{1}^{3}(x;6) \Psi_{2}^{3}(x;6)}>0 \end{aligned}$$

and

3.2 $$\begin{aligned} G''(x)=\frac{B(x-1)}{10 x^{3} (1 + x)^{3} (1 + 2 x)^{4} (11 + 28 x ^{2})^{3} (39 + 56 x + 28 x^{2})^{3}}< 0, \end{aligned}$$

where

$$\begin{aligned}& \Psi_{1}(x;6)=1{,}994{,}085{,}029{,}084{,}366 - 135{,}359{,}702{,}133{,}051 x^{2} \\& \hphantom{\Psi_{1}(x;6)={}}{}+ {914},{085},{135},{478},{944} x^{4}+ 376{,}162{,}173{,}891{,}504 x^{6}, \\& \Psi_{2}(x;6)=3{,}148{,}972{,}636{,}321{,}763 + 5{,}642{,}594{,}180{,}998{,}698 x +\cdots \\& \hphantom{\Psi_{2}(x;6)={}}{}+ 376{,}162{,}173{,}891{,}504 x^{6}. \end{aligned}$$

$A(x)=471{,}110{,}623{,}493{,}199{,}298{,}560 x^{20}+\cdots$ is a polynomial of 20th degree with all positive coefficients and $B(x)=-26{,}572{,}808{,}192 x^{12}-\cdots$ is a polynomial of 12th degree with all negative coefficients.

This shows that $F(x)$ is strictly convex and $G(x)$ is strictly concave on $(0,\infty )$. According to Theorem 1, when $n\rightarrow \infty $, it holds that $\lim_{n\rightarrow \infty }f(n) = \lim_{n\rightarrow \infty }g(n) = 0$; thus $\lim_{n\rightarrow \infty }F(n) = \lim_{n\rightarrow \infty }G(n) = 0$. As a result, we can make sure that $F(x) > 0$ and $G(x) < 0$ on $(0,\infty )$. Consequently, the sequence $f(n)$ is strictly increasing and $g(n)$ is strictly decreasing while they both converge to 0. As a result, we conclude that $f(n) < 0$ and $g(n) > 0$ for every integer ${n > 1}$.

The proof of Theorem 2 is complete.

## Numerical computations

In this section, we give Table 1 to demonstrate the superiority of our new series respectively. From what has been discussed above, we found out the new asymptotic function as follows:

4.1 $$\begin{aligned} n!\sim \sqrt{2\pi n} \biggl( \frac{n}{e} \biggr) ^{n} \exp \biggl( \frac{1}{12} \psi '(n+1/2) \biggr) \exp \bigl( \eta_{1}(n)+\eta_{2}(n) \bigr) = \beta (n), \end{aligned}$$

where

$$ \eta_{1}(n)=\frac{\frac{1}{240}}{n^{3}+\frac{11}{28}n}, \quad\quad \eta_{2}(n)= \frac{ \frac{193}{282{,}240}}{n^{7} + \frac{108{,}338}{44{,}583} n^{5} - \frac{21{,}252{,}897{,}179}{59{,}061{,}418{,}416} n^{3} + \frac{997{,}042{,}514{,}542{,}183}{188{,}081{,}086{,}945{,}752} n}. $$

**Table 1:** **Simulations for** $\pmb{\alpha_{1}(n)}$ **,** $\pmb{\alpha_{2}(n)}$ **and** $\pmb{\beta (n)}$

***n***	$\boldsymbol{\frac{\alpha _{1}(n)-n!}{n!}}$	$\boldsymbol{\frac{\alpha _{2}(n)-n!}{n!}}$	$\boldsymbol{\frac{\beta (n)-n!}{n!}}$
50	9.7924 × 10^−19^	1.1997 × 10^−24^	−7.0866 × 10^−28^
500	9.8013 × 10^−28^	1.2019 × 10^−37^	−7.1217 × 10^−43^
1000	1.9143 × 10^−30^	1.4672 × 10^−41^	−2.1734 × 10^−47^
2000	3.7389 × 10^−33^	1.7910 × 10^−45^	−6.63 × 10^−52^

Mortici and Qi [1] gave the formula

4.2 $$\begin{aligned} \begin{aligned}[b] n!&\sim \sqrt{2\pi n} \biggl( \frac{n}{e} \biggr) ^{n} \exp \biggl( \frac{1}{12} \psi '(n+1/2) \biggr) \exp \biggl( \frac{1}{240n^{3}}-\frac{11}{6720n ^{5}}+\frac{107}{80{,}640n^{7}} \biggr) \\ & = \alpha_{1}(n). \end{aligned} \end{aligned}$$

We can get the approximation by truncation of the asymptotic formula (1.9)

4.3 $$\begin{aligned} n!&\sim \sqrt{2\pi n} \biggl( \frac{n}{e} \biggr) ^{n} \exp \biggl( \frac{1}{12} \psi '(n+1/2) \biggr) \\ &\quad{}\times \exp \biggl( \frac{1}{240n^{3}}-\frac{11}{6720n^{5}}+ \frac{107}{80{,}640n ^{7}}-\frac{2911}{1{,}520{,}640n^{9}}+\frac{808{,}733}{184{,}504{,}320n^{11}} \biggr) \\ &= \alpha_{2}(n). \end{aligned}$$

The great advantage of our approximation $\beta (n)$ consists in its simple form and its accuracy. From Table 1, we can see that the formula $\beta (n)$ converges faster than the approximation of the formula $\alpha_{1}(n)$ and $\alpha_{2}(n)$.
